# Draft Genome Sequence of *Pseudomonas* sp. Strain In5 Isolated from a Greenlandic Disease Suppressive Soil with Potent Antimicrobial Activity

**DOI:** 10.1128/genomeA.01251-15

**Published:** 2015-11-25

**Authors:** Rosanna C. Hennessy, Mikkel A. Glaring, Charlotte F. Michelsen, Stefan Olsson, Peter Stougaard

**Affiliations:** a Department of Plant and Environmental Sciences, University of Copenhagen, Frederiksberg, Denmark; b Department of Systems Biology, Technical University of Denmark, Kgs. Lyngby, Denmark

## Abstract

*Pseudomonas* sp. In5 is an isolate of disease suppressive soil with potent activity against pathogens. Its antifungal activity has been linked to a gene cluster encoding nonribosomal peptide synthetases producing the peptides nunamycin and nunapeptin. The genome sequence will provide insight into the genetics behind the antimicrobial activity of this strain.

## GENOME ANNOUNCEMENT

*Pseudomonas* spp. are a rich source of secondary metabolites, including bioactive nonribosomal peptides (NRPs) and polyketides (1). NRPs are synthesized in large assembly lines by multidomain modular enzymes known as NRP synthetases (NRPS). Nunamycin and nunapeptin are two cyclic NRPs synthesized by the Greenlandic isolate *Pseudomonas* sp. In5. Nunamycin shows antifungal activity against the basidiomycete *Rhizoctonia solani*, whereas nunapeptin appears most active against the ascomycete *Fusarium graminearum* and the oomycete *Pythium aphanidermatum* (2). Originally isolated from disease suppressive soil from a potato field in Inneruulalik, South Greenland, *Pseudomonas* sp. In5 is therefore a promising potential biocontrol agent against plant pathogens (3, 4). In this report, we describe the annotated draft genome sequence of strain In5, which is part of ongoing research into antimicrobial secondary metabolites and novel biocontrol agents.

The genomic DNA of *Pseudomonas* sp. In5 was isolated from cultures growing in liquid medium. The draft genome was obtained by a combination of paired-end sequencing of a short-insert (500-bp) library and mate-pair sequencing of a large-insert (5-kb) library on an Illumina platform. Quality trimming of sequences and *de novo* assembly were performed using CLC Genomics Workbench version 7.5.1 (CLC bio, Qiagen, Aarhus, Denmark). The assembly resulted in 56 contigs organized in 18 scaffolds covering 7,318,798 bp, and almost the entire genome (99.9%) was covered by 5 large scaffolds. The G+C content was 59.4%. Genome annotation was performed using the NCBI Prokaryotic Genome Annotation Pipeline and identified 6,236 protein-coding sequences (CDSs) and 66 RNAs. antiSMASH (5) analysis of the genome identified 9 putative secondary metabolite gene clusters, including two NRPS clusters that generate the cyclic peptides nunamycin and nunapeptin, which were recently shown to possess both antimicrobial (2) and anticancer activities (C. F. Michelsen and P. Stougaard, unpublished data). Further in-depth analysis of this genome will increase our understanding of the role and regulation of In5 secondary metabolites during microbial interactions.

### Nucleotide sequence accession numbers.

This whole-genome shotgun project has been deposited at DDBJ/EMBL/GenBank under the accession no. LIRD00000000. The version described in this paper is the first version, LIRD01000000.

## References

[1] Gross H, Loper JE 2009 Genomics of secondary metabolite production by *Pseudomonas* spp. Nat Prod Rep 26:1408–1446. doi:10.1039/b817075b. 19844639

[2] Michelsen CF, Watrous J, Glaring MA, Kersten R, Koyama N, Dorrestein PC, Stougaard P 2015 Nonribosomal peptides, key biocontrol components for *Pseudomonas fluorescens* In5, isolated from a Greenlandic suppressive soil. mBio 6(2):e00079-15. doi:10.1128/mBio.00079-15. 25784695 PMC4453515

[3] Michelsen CF, Stougaard P 2011 A novel antifungal *Pseudomonas fluorescens* isolated from potato soils in Greenland. Curr Microbiol 62:1185–1192. doi:10.1007/s00284-010-9846-4. 21165740

[4] Michelsen CF, Stougaard P 2012 Hydrogen cyanide synthesis and antifungal activity of the biocontrol strain *Pseudomonas fluorescens* In5 from Greenland is highly dependent on growth medium. Can J Microbiol 58:381–390. doi:10.1139/w2012-004. 22417387

[5] Weber T, Blin K, Duddela S, Krug D, Kim HU, Bruccoleri R, Lee SY, Fischbach MA, Müller R, Wohlleben W, Breitling R, Takano E, Medema MH 2015 antiSMASH 3.0—a comprehensive resource for the genome mining of biosynthetic gene clusters. Nucleic Acids Res 43:W237–W243. doi:10.1093/nar/gkv437. 25948579 PMC4489286

